# Method of Finding the Dew-Point

**Published:** 1835

**Authors:** 


### XII.—Method of finding the Dew-point.

The gentleman quoted in the preceding articles, writes us from Philadelphia:

"I have learned from Mr. Espy, of this city, a very easy and convenient method of ascertaining the *dew-point.* As it originated with him, and has not been published, it may be interesting to you. I think you will find in Ure's Dictionary of Chemistry, tables by which the humidity of the atmosphere, etc., can be ascertained whenever the dew-point is known. It is troublesome to ascertain the dew-point by the usual method. Suppose, therefore, the thermometer is at 79°, and another thermometer with the bulb wetted or wrapped with a wet cloth is at 67°5. The dew-point will be found to be 61°5, by the following method: 79°—67°.5=11,5 × 103 =1184,5÷67,5=61,5. The 103 is a fixed term, arrived at by experimental labor. It is necessary, in order to arrive at this result, that the thermometer as well as the wet bulb thermometer, should be fanned for ten or fifteen minutes, and to take the numbers from what the two thermometers indicate after the process of ventilation. For instance, the thermometer might have stood at 80° or 81° in a still atmosphere, but by fanning the air around it, it would fall to 79°. By knowing the dew-point at any time, it will be an easy matter to tell how much vapor the lungs are throwing off—whether the patient be in too humid an atmosphere, or not sufficiently so—and many things of the kind which may be turned to practical account."

